# Supplementary material for: Therapeutic homology-independent targeted integration in retina and liver
Source: Nat Commun. 2022 Apr 12;13:1963. doi: 10.1038/s41467-022-29550-8 (PMC9005519; doi:10.1038/s41467-022-29550-8)
Supplement: Supplementary file 1 — Supplementary Information [file 41467_2022_29550_MOESM1_ESM.pdf]

Supplementary Information for
Therapeutic homology-independent targeted integration in retina and liver

Patrizia Tornabene[1,2#], Rita Ferla[1,2#], Manel Llado-Santaeularia[1#], Miriam Centrulo[1], Margherita Dell'Anno[1,2], Federica Esposito[1], Elena Marrocco[1], Emanuela Pone[1,2], Renato Minopoli[1], Carolina Iodice[1], Edoardo Nusco[1], Settimio Rossi[3], Hristiana Lyubenova[1], Anna Manfredi[4,5], Lucio Di Filippo[5], Antonella Iuliano[1], Annalaura Torella[1,6], Giulio Piluso[6], Francesco Musacchia[1], Enrico Maria Surace[2], Davide Cacchiarelli[4,7], Vincenzo Nigro[1,6] and Alberto Auricchio[1,8*]

Affiliations:

1. Telethon Institute of Genetics and Medicine (TIGEM), Pozzuoli 80078, Italy.
2. Medical Genetics, Department of Translational Medicine, Federico II University, Naples 80131, Italy.
3. Eye Clinic, Multidisciplinary Department of Medical, Surgical and Dental Sciences, University of Campania L. Vanvitelli, Naples 80131, Italy.
4. Telethon Institute of Genetics and Medicine (TIGEM), Armenise/Harvard Laboratory of Integrative Genomics, Pozzuoli 80078, Italy.
5. Next Generation Diagnostic srl, Pozzuoli 80078, Italy.
6. Department of Precision Medicine, University of Campania L. Vanvitelli, Naples, 80138, Italy.
7. Department of Translational Medicine, Federico II University, Naples 80131, Italy.
8. Medical Genetics, Department of Advanced Biomedical Sciences, Federico II University, Naples 80131, Italy.

#Contributed equally
*Correspondence should be addressed to: Alberto Auricchio, M.D.
Telethon Institute of Genetics and Medicine (TIGEM), Pozzuoli 80078, Italy.
Medical Genetics, Department of Advanced Biomedicine, Federico II University, Naples 80131, Italy.
E-mail: auricchio@tigem.it

TABLE OF CONTENTS

SUPPLEMENTARY RESULTS AND DISCUSSION 3

In vitro testing of gRNAs and donor DNA integration 3
Molecular characterization of HITI efficiency, precision and safety in the retina 3
The IRES START site results in leaky DsRed expression in hepatocytes 5
Molecular characterization of HITI efficiency, precision and safety in the liver 5

SUPPLEMENTARY FIGURES 7

Fig. Supplementary 1. Cleavage efficiency of gRNAs and HITI efficiency in vitro. 7
Fig. Supplementary 2. Schematic representation of the AAV genomes from plasmids used in Figures 2, Supplementary Figure 3-5 and Supplementary Table 3. 8
Fig. Supplementary 3. Safety, efficiency and precision of HITI in mouse photoreceptors. 10
Fig. Supplementary 4. Specific SpCas9 cleavage and HITI efficiency in pig photoreceptors. 11
Fig. Supplementary 5. In vitro expression of hRHO and morphological improvement in treated P23H mice. 12
Fig. Supplementary 6. Schematic representation of the AAV plasmids for generation of vectors used in Figure 3, Supplementary Figures 7 and 8 and Supplementary Table 5. 13
Fig. Supplementary 7. The inclusion of IRES as translation start site in the donor DNA results in SpCas9-independent expression of DsRed. 14
Fig. Supplementary 8. Safety and efficiency of HITI in mouse hepatocytes. 16

SUPPLEMENTARY TABLES 17

Table Supplementary 1: gRNAs and scramble sequences used to target SpCas9 to the rhodopsin and albumin loci in different species 17
Table Supplementary 2. Top 10 ranked OFF-targets of mRho gRNA 18
Table Supplementary 3. Predicted genotype frequency following HITI at the mRho locus. 19
Table Supplementary 4. Top 10 ranked OFF-targets of mAlb gRNA 20
Table Supplementary 5: Genome-wide distribution of donor DNA integration 21

SUPPLEMENTARY REFERENCES 22

UNCROPPED IMAGES 25

Uncropped images of Supplementary Figure 3c 25
Uncropped image of Supplementary Figure 4a 26
Uncropped images of Supplementary Figure 8c 27

Supplementary Results and Discussion

In vitro testing of gRNAs and donor DNA integration

We assessed the efficiency of guide RNAs (gRNAs, Table Supplementary 1) targeting mouse *rhodopsin* (m*Rho*), pig *Rhodopsin* (p*RHO*) and mouse *albumin* (m*Alb*) *in vitro* by Tracking of insertions/deletions (INDELs) by decomposition (TIDE) analysis and found similar (p= 0,87) levels of INDELs while no INDELs were found in scramble-treated cells (Fig. Supplementary 1a).
To check proper integration and expression of *Discosoma red fluorescent protein* (DsRed), we generated two donor DNAs with different translation start sites (STARTs): one with kozak (kozak-DsRed) and the other with the internal ribosomal entry site[1] (IRES, IRES-DsRed, Fig. Supplementary 1b).These donor DNAs were tested *in vitro* in HEK293 cells, using a plasmid encoding m*Rho* P23H under the control of the cytomegalovirus (CMV) promoter (CMV-m*Rho* P23H) as a template for integration and expression of DsRed (Fig. Supplementary 1b). Independently of the START used, fluorescence-activated cell sorting (FACS) analysis showed that in gRNA-treated cells, most DsRed+ cells detected were also positive to *enhanced green fluorescent protein* (EGFP) co-expressed with *Streptococcus pyogenes* Cas9, SpCas9 (Fig. Supplementary 1c). We then quantified HITI efficiency on sorted DsRed+/EGFP+ cells ratio and observed 48,1% of DsRed+/EGFP+ cells when using kozak-DsRed donor DNA and 24,8% when using IRES-DsRed (Fig. Supplementary 1c).

Molecular characterization of HITI efficiency, precision and safety in the retina

For a comprehensive evaluation of homology-independent targeted integration (HITI) *in vivo*, we characterized its efficiency, precision and safety in the retina. First, we quantified INDEL frequency at the targeted m*Rho* locus, which was about 3% using either TIDE or next generation sequencing (NGS) (Fig. Supplementary 3a, b). We also measured INDEL frequency for the top 10 predicted off-target sites of the m*Rho*-gRNA (Table Supplementary 2). Sites 1 and 5 showed slightly higher INDEL frequencies (0,6 and 0,3%, respectively) in gRNA- than in scramble-treated retina (Fig. Supplementary 3b). Site 1 represents an intronic region of the *Nsmce2* gene, which has been described to be involved in DNA double-strand break repair, acting as a tumor suppressor in several tissues including the retina[3]. Site 5 represents an intronic region of the *Mrtfb* gene, a myocardin-related transcription factor that is not reported to be expressed in the retina[4] and is described to be involved in gene expression regulation and neuronal morphology[5]. INDELs in both off-target sites should not disrupt the expression of the corresponding genes as they occur in intronic regions. Although this gRNA will not be used for therapeutic purposes in humans since it is specific for a mouse gene, the relatively high INDEL frequencies at sites 1 and 5 highlight the need for a thorough gRNA characterization before further translating Cas9-based approaches. We also looked for potential large gene rearrangements and found no presence of PCR-detectable insertions or deletions up to 9kb upstream or downstream of the cleavage site in gRNA-treated retina (Fig. Supplementary 3c) with the exception of the expected insertion of the donor DNA (Fig. Supplementary 3c), suggesting that rearrangements with a low frequency are unlikely to be detected by this PCR method.
In order to define the frequency of HITI at the m*Rho* locus compared to m*Rho* alleles without HITI, we produced similar-size PCR fragments using a forward primer that anneals to the m*Rho* sequence and two reverse primers, one that anneals to m*Rho* and the other to DsRed on the donor DNA. NGS analysis of these PCR products showed 5% of reads that contained specific integration of the donor DNA allele, 9% INDELs in m*Rho,* and the remaining 86% had the wild-type *Rho* sequence (Fig.

Supplementary 3d). The percentage of HITI observed by NGS is similar to the percentage of DsRed+ photoreceptors counted on retinal cryosections, while the presence of m*Rho* without HITI with INDELs suggests that increasing availability of the donor DNA could further increase HITI efficiency. In order to define the genotypes produced by AAV-HITI in $Rho^{P23H-/+}$ mice, we have applied the Hardy Weinberg equation to the allele frequencies presented in Figure Supplementary 3d, and obtained the expected genotypic outcomes (Table Supplementary 3): 74% of cells will contain the unedited P23H/wild-type genotype; of the remaining 26% edited cells: 13,2% will contain either the INDEL/wild-type or HITI/wild-type or INDEL/HITI or HITI/HITI genotypes, all expected to be therapeutic; 4,3% will be compound heterozygous for P23H/HITI which are predicted to be similar to the original P23H/wild-type genotype of our mouse model (therefore defined as "no effect"); 7,7% will be P23H/INDEL which based on a dominant negative effect of the P23H mutation[6] is expected to behave similarly to the original P23H/wild-type genotype (therefore defined as "presumably no effect"); 0,8% will be INDEL/INDEL and therefore defined as "deleterious" based on the severity of the $Rho^{-/-}$ knock-out mouse model retinal phenotype[7–9] compared to the phenotype of the $Rho^{P23H-/+}$ mouse model. Therefore, only a minority of the genotypic outcomes of HITI will be deleterious with a significantly larger fraction being therapeutic (Table Supplementary 3).
Importantly, since HITI relies on non-homologous end joining (NHEJ) to achieve targeted integration, INDELs can be generated at the ends of the donor DNA during integration[10,11]. Those INDELs could potentially affect the expression of the therapeutic coding sequence. In order to characterize the precision of HITI in the m*Rho* locus, we amplified HITI junctions (Fig. Supplementary 3e) and performed NGS analysis to identify and quantify the position, type and frequency of the INDELs generated. A low frequency of insertions (4,6%) and deletions (13,6%) was observed at the 5' junction, especially around the SpCas9 cleavage site (Fig. Supplementary 3e). Interestingly, we observed two insertions of 40 bp and 48 bp which were compatible with partial integration of AAV inverted terminal repeats in the DNA double-strand breaks. The most common insertion was 1 bp (1,9%) while deletions ranging from 1 to 9 bp always localized upstream of the cleavage site, thus outside of the donor DNA (Fig. Supplementary 3e). Therefore, approximately 80% of reads showed INDEL-free HITI. At the 3' junction, we observed a surprisingly high (62,2%) rate of 1 bp insertions (Fig. Supplementary 3e), which was significantly higher than expected[10]. We also observed a fairly common (5,5%) 2 bp insertion (Fig. Supplementary 3e). However, these insertions at the 3' of our donor DNA did not affect the coding sequence and thus are not predicted to have deleterious effects. Deletions at the 3' junction were infrequent, with 2,1% of 3 bp deletions and 3% of 6 bp deletions. In total, around 70% of reads at the 3' junction presented INDELs, mostly found around the cleavage site (Fig. Supplementary 3e).
Finally, the analysis of samples from the pig retina transduced with AAV-HITI allowed us to confirm that SpCas9 cleavage of the p*RHO* locus only occurred in the neural retina and not in the retinal pigmented epithelium (Fig. Supplementary 4a), as expected based on the photoreceptor-specific expression of SpCas9 from the interphotoreceptor retinol-binding protein promoter. Interestingly, the cleavage efficiency observed by TIDE and NGS in the pig retina (19% and 17% respectively, Fig. Supplementary 4b-c) is higher than that observed in the mouse retina. This could be due to the higher level of AAV co-transduction in pig compared to mouse photoreceptors. However, this did not have a significant effect in the efficiency of HITI, which was 4% in the pig retina (Fig. Supplementary 4c), similar to that observed in mouse (Fig. Supplementary 3d). This confirms our assessment that co-transduction is not the only limiting factor on HITI efficiency.

The IRES START site results in leaky DsRed expression in hepatocytes

To test IRES in hepatocytes, AAV vectors carrying an IRES-DsRed with either the gRNA or the scramble expression cassette (Fig. Supplementary 6b) were injected in combination with an AAV vector expressing SpCas9 under the hybrid liver specific promoter (HLP-SpCas9, Fig. Supplementary 6a) in neonatal C57BL/6J mice at the same doses described for the kozak constructs. Unexpectedly, both gRNA- and scramble-treated livers collected at post-natal day 30 showed presence of DsRed+ hepatocytes, which was higher in gRNA-treated livers compared to scramble controls (Fig. Supplementary 7a). INDELs were only detected in gRNA- (14,2%) but not in scramble-treated livers (Fig. Supplementary 7b). The unexpected expression of DsRed when using IRES could be due to integration of the whole AAV genome in double strand breaks in the hepatocyte genome, since IRES should have a better ability than kozak to drive expression of DsRed from neighboring promoters. For this reason, we exclusively used the kozak as START site in the experiments described in Figure 3 and Supplementary Figure 8.

Molecular characterization of HITI efficiency, precision and safety in the liver

We then characterized the efficiency, precision and safety of HITI in the liver, similarly to what we had previously done in the retina. First, we used TIDE to quantify INDEL frequency at the targeted *albumin* locus, which was about 10% (Fig. Supplementary 8a). Interestingly, INDEL frequency was similar at the three time points tested (Fig. Supplementary 8a). The frequency of INDELs, which does not increase over time as expected, supports the loss of SpCas9 expression due to AAV genome dilution during hepatocyte proliferation. We then used NGS to quantify INDELs at both the on-target and the top 10 predicted off-target sites of the m*Alb*-gRNA (Table Supplementary 4). No difference in INDEL frequency was observed between gRNA and scramble samples at any of the off-target sites (Fig. Supplementary 8b). INDELs on-target at m*Alb* had a frequency of 21% in gRNA samples (Fig. Supplementary 8b).
As in the retina, we looked for potential large chromosomal rearrangements and found no presence of PCR-detectable insertions or deletions up to 9kb upstream or downstream the cleavage site in gRNA -treated samples, except for the expected donor DNA integration (Fig. Supplementary 8c).
Next, we performed NGS analysis of donor DNA integration at the m*Alb* locus. In gRNA- but not in scramble- treated livers, 4% of total reads which covered the target site contained integration of the donor DNA (Fig. Supplementary 8d). This is consistent with the efficiency observed by DsRed fluorescence (2%), and the difference can be attributed to bi-allelic editing potentially occurring in some hepatocytes.
We then evaluated HITI precision by NGS analysis of HITI junctions (Fig. Supplementary 8e). At the 5’ junction we found that about 4% of total reads had insertions and 36% had deletions (Fig. Supplementary 8e). At the 3’ junction, instead, we observed a high frequency of 1 bp insertions (15%) near to the cleavage site, as well as some 2 bp insertions (1,5%, Fig. Supplementary 8e). Deletions were less frequent (13%) than those found at the 5’ junction (Fig. Supplementary 8e). A 42 bp deletion with low frequency (0,4%) was identified upstream of the double-strand breaks at the 3’ junction. Importantly, on-target integration in the liver was almost as precise as expected[10] with 60-70% INDEL-free integration. While deletions and insertions observed at the 5’ junction were not expected to alter expression of the donor DNA, large (>20bp) deletions at the 3’ junction might result in partial deletion of the bovine growth hormone polyadenylation (pA) sequence. To avoid this, we added a 200 bp stuffer DNA between the pA and the gRNA target site in the therapeutic donor DNA (Fig. Supplementary 6c) used in the experiments described in Figure 3b-i.

Finally, to characterize potential HITI off-targets, we used a genome-wide approach. HITI off-targets were expected to be observed in gRNA- but not in scramble-treated livers. In DNA samples extracted from gRNA-treated livers, integration of the donor DNA in m*Alb* was observed in 13% of the total reads, while only in 0,24% of total reads obtained in scramble samples (Fig. Supplementary 8f, Table Supplementary 5). We did not observe any other locus in the genome that showed selective donor integration in gRNA- but not in scramble-treated livers. Interestingly, we did observe some loci in which donor DNA integration was present in both gRNA- and scramble-treated livers, suggesting integration of AAV fragments in SpCas9-independent, spontaneous DNA double-strand breaks. Most integrations were localized to chromosomes 2, 3, 11, 13 and 19 (Fig. Supplementary 8f, Table Supplementary 5). Out of 36 off-target integrations identified, 17 were intergenic. Of the 19 intragenic integrations, 4 were found at low frequency in exons of the following genes: *Afp* encoding alpha-fetoprotein, *Trf* encoding transferrin and *Kmt2d* encoding lysine (K)-specific methyltransferase 2D. *Afp* and *Trf* are both highly expressed in liver, which is consistent with preference of AAV for integration in active genes[12]. Indeed, AAV integrations in *Alb* and *Trf* following neonatal gene transfer have been previously reported, however without correlation with hepatocellular carcinoma (HCC) development. *Kmt2d* has been associated with Kabuki syndrome[13] and found to be mutated in several tumors including HCCs[14,15]. However, to the best of our knowledge, HCCs with AAV integration in this gene have not been reported so far. Of the loci with intronic AAV integration, 5 have been found in genes (*Ctnap5b, Asic2, Erc2, Eml6, Ugt1A10*) variously implicated in human tumors but not in HCCs[16–21]. Low frequency AAV integrations were also found in *Alcam* (encoding the activated leukocyte cell adhesion molecule) which is involved in HCC migration and invasion[22–24] and *L3mbtl4* (encoding L3MBTL Histone Methyl- Lysine Binding Protein 4) which has been reported both as a susceptibility locus for Hepatitis B virus-induced HCC and as a tumor suppressor in breast cancer[25]. Finally, we found the highest frequency (15%) of HITI-independent AAV integrations in *Prdm12* encoding the positive regulatory (PR)-domain zinc-finger protein 12 and *Dpp6* encoding the Dipeptidyl Peptidase Like 6. Altered expression of both these genes has been found in HCC[26,27], although neither mutations in these genes nor other evidence of their direct role in HCC development have been reported, though evidence of this nature has been reported for other tumors[28,29] (Table Supplementary 5). Thus, our data show that AAV integration (not HITI) occurs in some loci with high gene activity during hepatocyte growth, as expected. Interestingly, while we confirm AAV integrations in already reported loci, other integration sites have not been described before and none of them have been reported to be associated with HCC thus suggesting their relatively safe profile. Among the AAV integrations observed, we did not identify the *Rian* locus, which has been shown to harbor AAV integration in mouse HCCs[30–32]. This is not surprising as integration in this locus is a rare event that becomes detectable in HCC DNA because of its clonal nature.

Overall, our data suggest that AAV-HITI is a precise on-target approach and does not generate relevant off-target integrations in both liver and retina, laying the groundwork for possible future applications of this technology.

Supplementary Figures

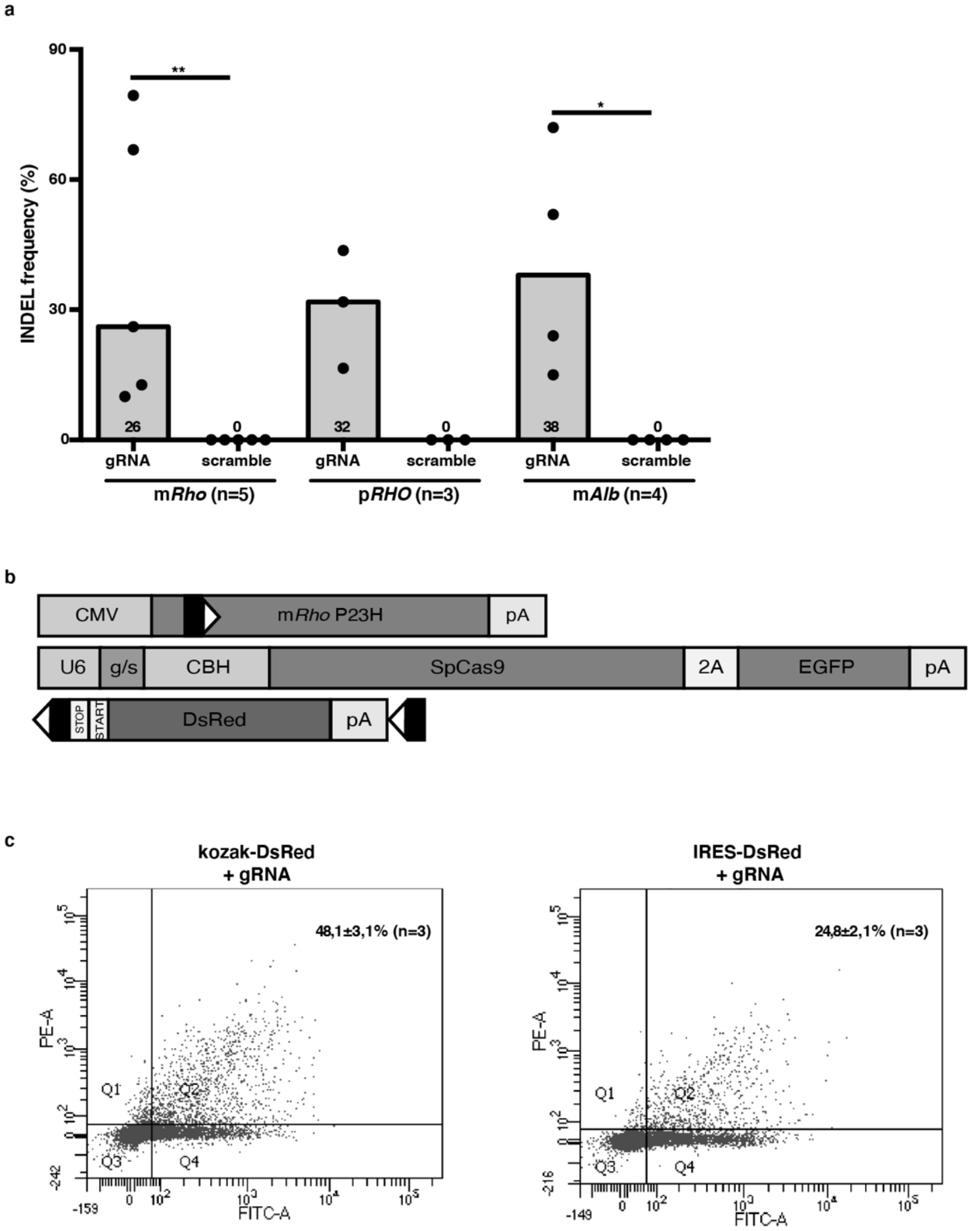

Fig. Supplementary 1. Cleavage efficiency of gRNAs and HITI efficiency in vitro.

(**a**) TIDE quantification of *in vitro* cleavage efficiency of the gRNAs used in this manuscript. (**b**) Plasmids used for transient transfection of HEK293 cells for *in vitro* testing of HITI in the m*Rho* locus; black rectangles and white triangles represent the two parts of the SpCas9 target sites flanking the SpCas9-induced double strand break. (**c**) Representative FACS graph of cells 48h after transfection with plasmids depicted in panel **b**; the percentage of EGFP+/DsRed+cells, is reported inside each graph. Results are represented as a single measurement for each experiment (dot) and as median for each group of treatment (column, **a**) or as mean ± SEM (panel **c**). *= p< 0,05; **= p<0,01 (**a**, Wilcoxon rank sum test; **c**, unpaired t-test). Further details on statistical analysis, including exact p value, can be found in Methods section. Source data are provided as a Source Data file. Abbreviations: m*Rho*: mouse *rhodopsin*; pRHO: pig *Rhodopsin*; mAlb: mouse *albumin*; CMV: Cytomegalovirus promoter; m*Rho* P23H: coding sequence for mouse *rhodopsin* (m*Rho*) bearing the P23H mutation; pA: bovine growth hormone polyA; U6: U6 promoter for the gRNA expression; g: gRNA specific for m*Rho*; s: scramble gRNA; CBH: Chicken beta hybrid promoter; SpCas9: *Streptococcus pyogenes* Cas9; 2A: ribosomal skipping sequence from Thosea asigna virus; STOP: stop codons in 3 open reading frames; START: translation start site (either kozak or internal ribosomal entry site, IRES); DsRed: *Discosoma red fluorescent protein*; Q1: EGFP-/DsRed+; Q2: EGFP+/DsRed+; Q3: EGFP-/DsRed-; Q4: EGFP+/DsRed-; PE-A: DsRed fluorescence filter; FITC-A: EGFP fluorescence filter; n: number of biologically independent samples.

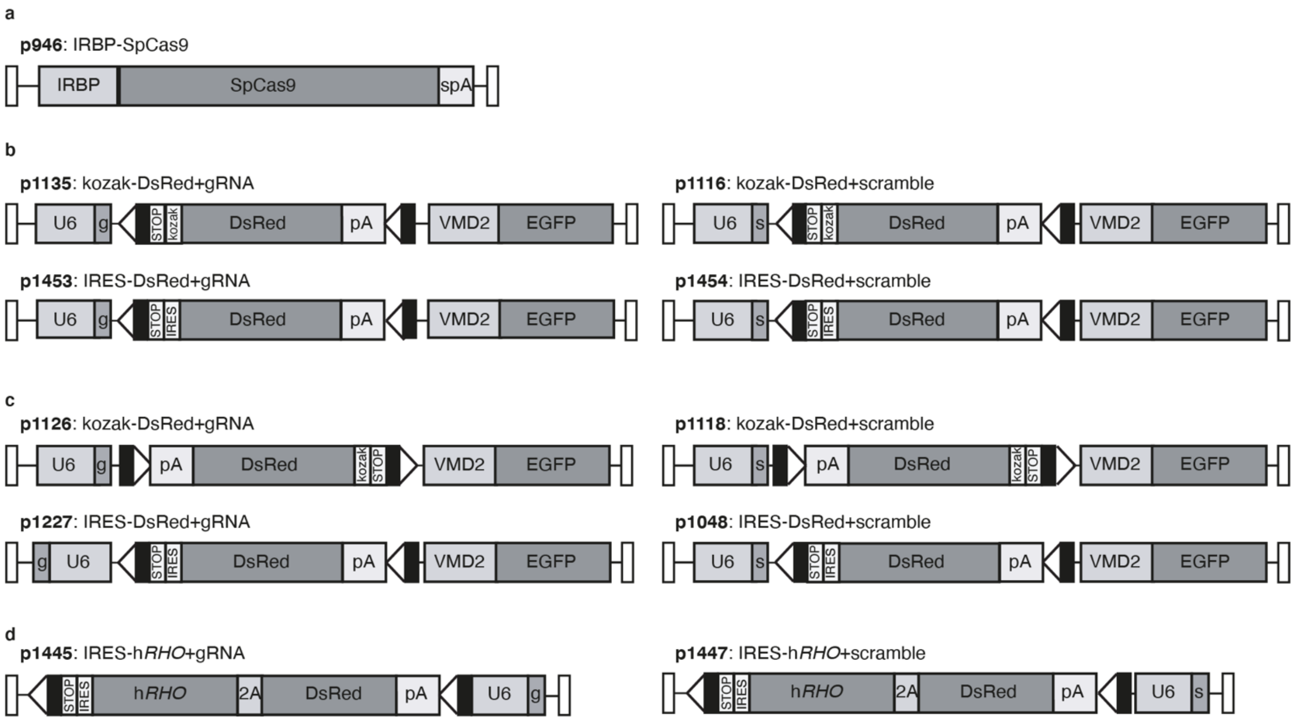

Fig. Supplementary 2. Schematic representation of the AAV genomes from plasmids used in Figures 2, Supplementary Figure 3-5 and Supplementary Table 3.

Schematic representation of the AAV genomes from the: AAV-IRBP-SpCas9-spA plasmid (**a**); AAV plasmids containing the donor DNAs used in either C57BL/6J mice (**b**), pigs (**c**) or *Rho*$^{P23H-/+}$ (**d**) mice. Inverted terminal repeats (ITRs) are depicted as white rectangles; black rectangles and white triangles represent the two parts of the SpCas9 target sites flanking the SpCas9-induced double strand break. Abbreviations: IRBP: interphotoreceptor binding protein promoter; SpCas9: *Streptococcus pyogenes* Cas9; spA: short synthetic polyadenylation sequence; U6: U6 promoter for gRNA expression; g: gRNA specific for either mouse *rhodopsin* (**b**, **d**) or pig *Rhodopsin* (**c**)*;* STOP: stop codons in 3 open reading frames; pA: bovine growth hormone polyadenylation sequence; DsRed: *Discosoma red fluorescent protein*; VMD2: vitelliform macular degeneration 2 promoter; EGFP: *enhanced green fluorescent protein*; s: scramble gRNA; IRES: internal ribosomal entry site; h*RHO*: coding sequence of the human *Rhodopsin* (h*RHO*); 2A: ribosomal skipping sequence from Thosea asigna virus.

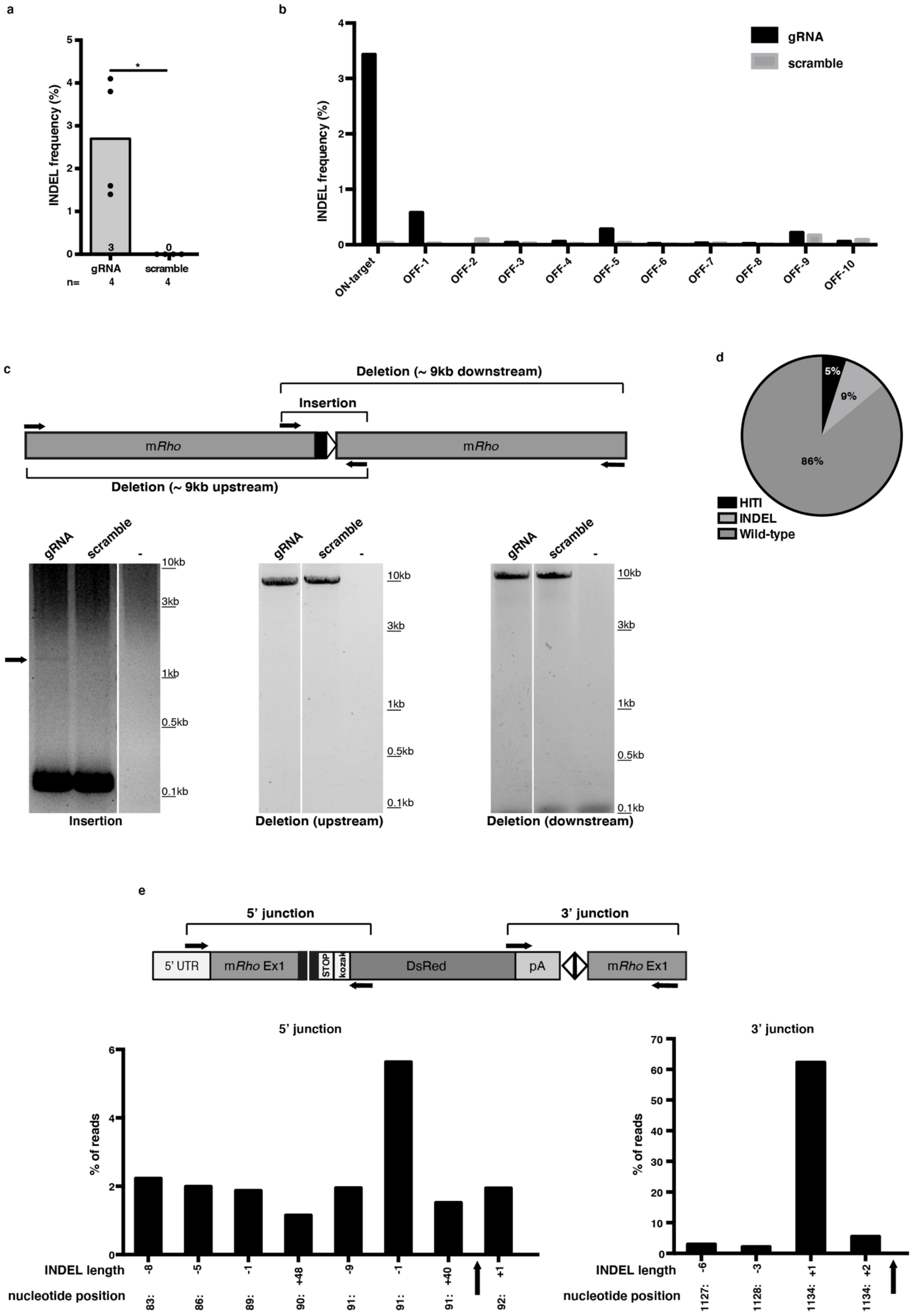
a
INDEL frequency (%)
*
gRNA
scramble
n=
4
4
b
INDEL frequency (%)
gRNA
scramble
ON-target
OFF-1
OFF-2
OFF-3
OFF-4
OFF-5
OFF-6
OFF-7
OFF-8
OFF-9
OFF-10
c
Deletion (~ 9kb downstream)
Insertion
mRho
mRho
Deletion (~ 9kb upstream)
gRNA
scramble
-
10kb
3kb
1kb
0.5kb
0.1kb
Insertion
Deletion (upstream)
Deletion (downstream)
d
5%
9%
86%
HITI
INDEL
Wild-type
e
5' junction
3' junction
5' UTR
mRho Ex1
STOP
kozak
DsRed
pA
mRho Ex1
% of reads
INDEL length
nucleotide position

Fig. Supplementary 3. Safety, efficiency and precision of HITI in mouse photoreceptors.

(**a**) TIDE quantification of INDELs on DNAs extracted from injected retinas. (**b**) NGS quantification of INDEL frequency at the m*Rho* (ON-target) and at the 10 top off-target (OFF-1-10) sites for the m*Rho*-gRNA, in gRNA- and scramble-treated retina. (**c**) Characterization of large rearrangements by PCR. Schematic depiction of the location of the primers used for PCR amplification of potential large insertions and deletions after SpCas9 cleavage at the m*Rho* locus and relative representative PCR products obtained in 3 gRNA- and 2 scramble-treated samples. The arrow points out to the expected size of the donor DNA integration which was detected in 1 out of 3 gRNA-injected eyes. (**d**) NGS quantification of HITI and INDEL frequency in gRNA-treated retinas. (**e**) Characterization of HITI precision by NGS analysis of HITI junctions. Schematic depiction of the location of the primers used for PCR amplification of HITI junctions and relative INDEL frequency at each position surrounding the cleavage site of either the HITI 5' or 3' junction. Negative and positive numbers represent deletions and insertions, respectively. The black arrow points at the SpCas9-mediated double-strand break. Results are represented as a single measurement for each mouse (dot) and as median for each group of treatment (column, **a**-**b**) or as mean percentage of total reads (pie, **d**). *= p< 0,05 (**a**, Wilcoxon rank sum test). Further details on statistical analysis, including exact p value, can be found in Methods section. Source data are provided as a Source Data file. Abbreviations: 5'UTR: 5' untranslated region; m*Rho:* mouse *rhodopsin*; Ex: exon; STOP: stop codons in 3 open reading frames; DsRed: *Discosoma red fluorescent protein*; pA: bovine growth hormone polyadenylation sequence; black rectangles and white triangles represent the two parts of the SpCas9 target sites flanking the SpCas9-induced double strand break; n: number of biologically independent samples.

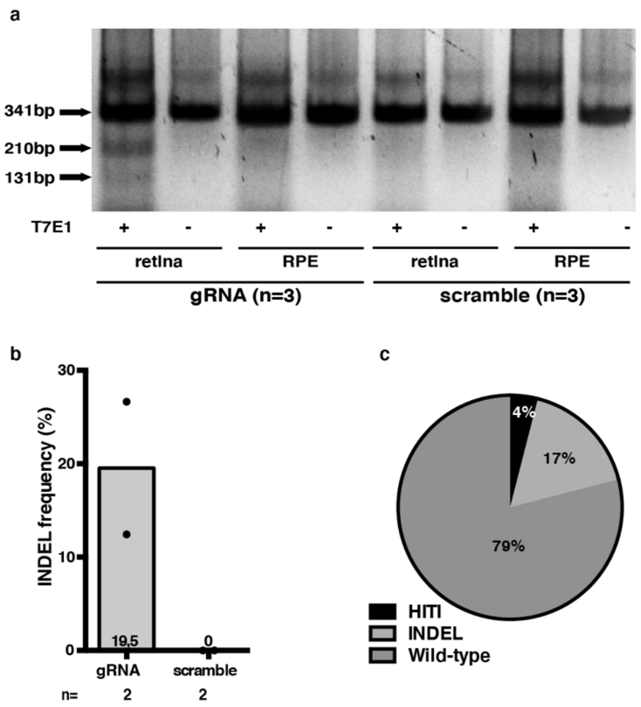

Fig. Supplementary 4. Specific SpCas9 cleavage and HITI efficiency in pig photoreceptors.

(**a**) T7E1 assay performed with DNA extracted from either retina or retinal pigmented epithelium (RPE) of injected eyes. The arrow points out the expected cleavage bands upon SpCas9-mediated double strand DNA break, which were detected in 2 out of 3 gRNA-injected eyes. (**b**) TIDE quantification of INDELs was performed on DNA extracted from injected retinas. (**c**) NGS quantification of HITI and INDEL frequency in gRNA-treated retinas. Results are represented as a single measurement for each mouse (dot) and as median for each group of treatment (column, **b**) or as mean percentage of total reads (pie, **c**). **b**, Wilcoxon rank sum test. Further details on statistical analysis, including exact p value, can be found in Methods section. Source data are provided as a Source Data file. Abbreviations: T7E1= T7 Endonuclease 1 treatment; n: number of biologically independent samples.

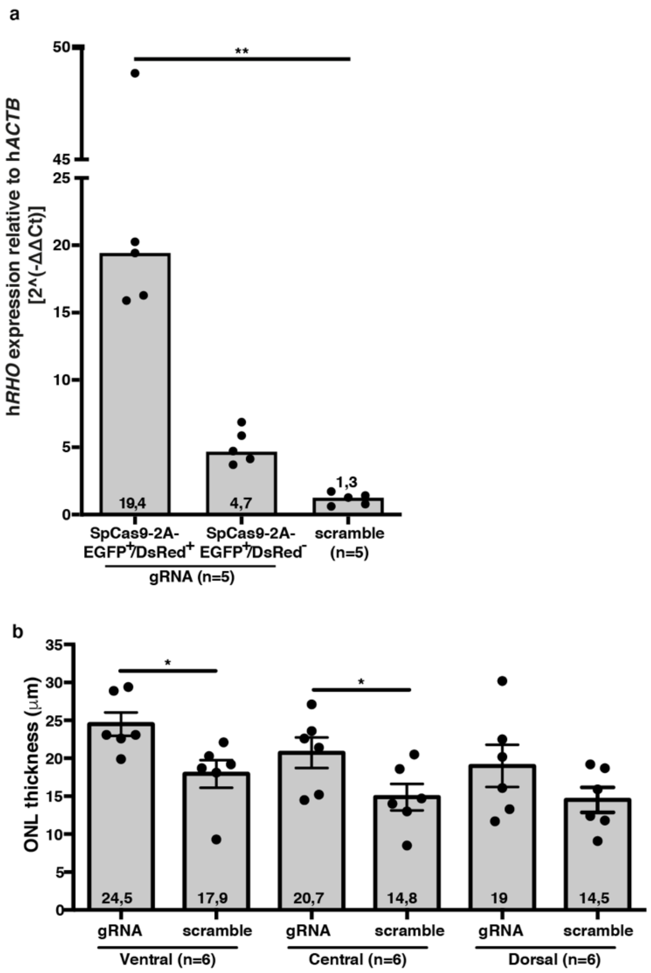

Fig. Supplementary 5. In vitro expression of hRHO and morphological improvement in treated P23H mice.

(**a**) h*RHO* expression following HITI in HEK293 cells was measured by Real-Time PCR. (**b**) Measurement of outer nuclear layer thickness in three retinal slices, from ventral to dorsal, representative of each whole eye. Results are represented as a single measurement for each experiment or mouse (dot, **a**-**b**) and as median for experiment (column, **a**) or as mean ± SEM for each group of treatment (column, **b**). *= p< 0,05; **= p< 0,01 (**a**, Kruskal-Wallis test followed by the Dunn's test; **b**, two-way ANOVA followed by Emmeans post-hoc test). Further details on statistical analysis, including exact p value, can be found in Methods section. Source data are provided as a Source Data file. Abbreviations: h*RHO:* human *Rhodopsin*; h*ACTB*: human β-*Actin* gene; SpCas9: *Streptococcus pyogenes* Cas9; 2A: ribosomal skipping sequence from Thosea asigna virus; EGFP: *enhanced green fluorescent protein*; DsRed: *Discosoma red fluorescent protein;* ONL: outer nuclear layer; n: number of biologically independent samples.

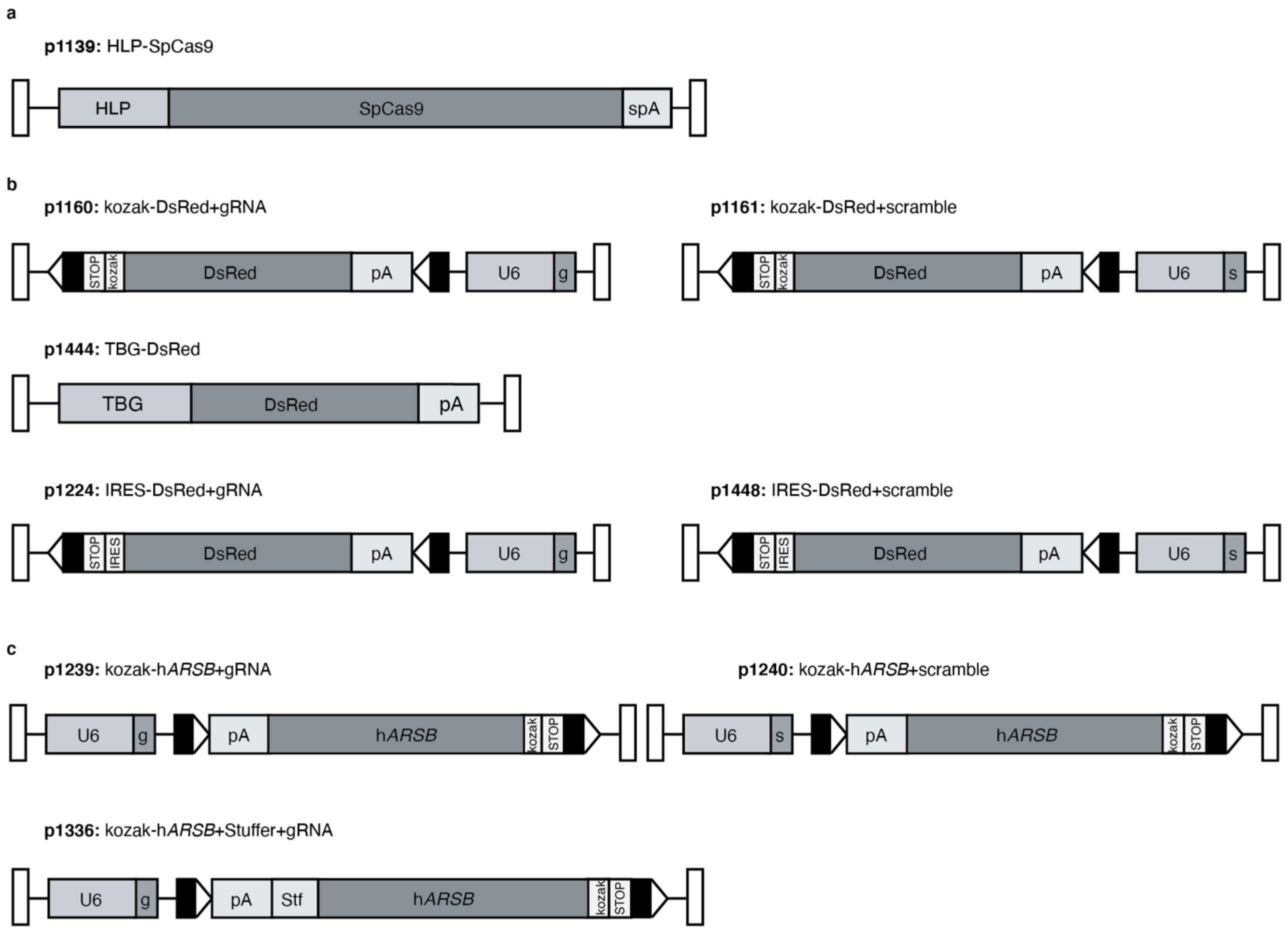

Fig. Supplementary 6. Schematic representation of the AAV plasmids for generation of vectors used in Figure 3, Supplementary Figures 7 and 8 and Supplementary Table 5.

Schematic representation of the AAV genomes from the: AAV-HLP-SpCas9-spA plasmid (**a**); AAV plasmids containing the donor DNAs used in either C57BL/6J mice (**b**), or MPS VI (**c**) mice. Inverted terminal repeats (ITRs) are depicted as white rectangles; black rectangles and white triangles represent the two parts of the SpCas9 target sites flanking the SpCas9-induced double strand break. Abbreviations: HLP: hybrid liver promoter; SpCas9: *Streptococcus pyogenes* Cas9; spA: short synthetic polyadenylation sequence; STOP: stop codons in 3 open reading frames; DsRed: *Discosoma red fluorescent protein* transgene; pA: bovine growth hormone polyadenylation sequence; U6: U6 promoter for gRNA expression g: gRNA specific for mouse *albumin* (m*Alb*); s: scramble gRNA; TBG: thyroxine-binding globulin promoter; IRES: internal ribosomal entry site; h*ARSB*: coding sequence of the human *Arylsulfatase B*; Stf: 200bp stuffer DNA.

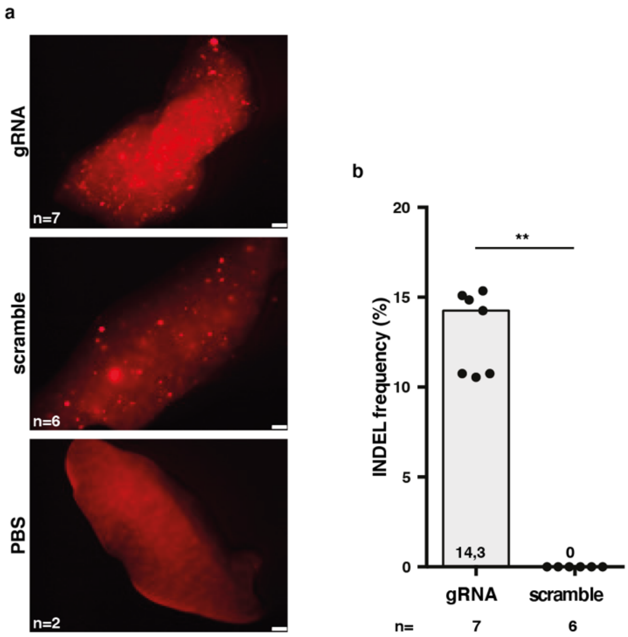

Fig. Supplementary 7. The inclusion of IRES as translation start site in the donor DNA results in SpCas9-independent expression of DsRed.

(**a**) Stereoscope fluorescence representative images showing DsRed+ foci in both gRNA and scramble- but not in PBS-treated livers. White scale bars equal 500 µm. (**b**) TIDE quantification of INDELs was performed on DNA extracted from treated livers. Results are presented as a single measurement for each liver (dot) and median for each group of treatment (column). ** = p<0,01 (**b**, Wilcoxon rank sum test). Further details on statistical analysis, including exact p value, can be found in Methods section. Abbreviations: n: number of biologically independent samples.

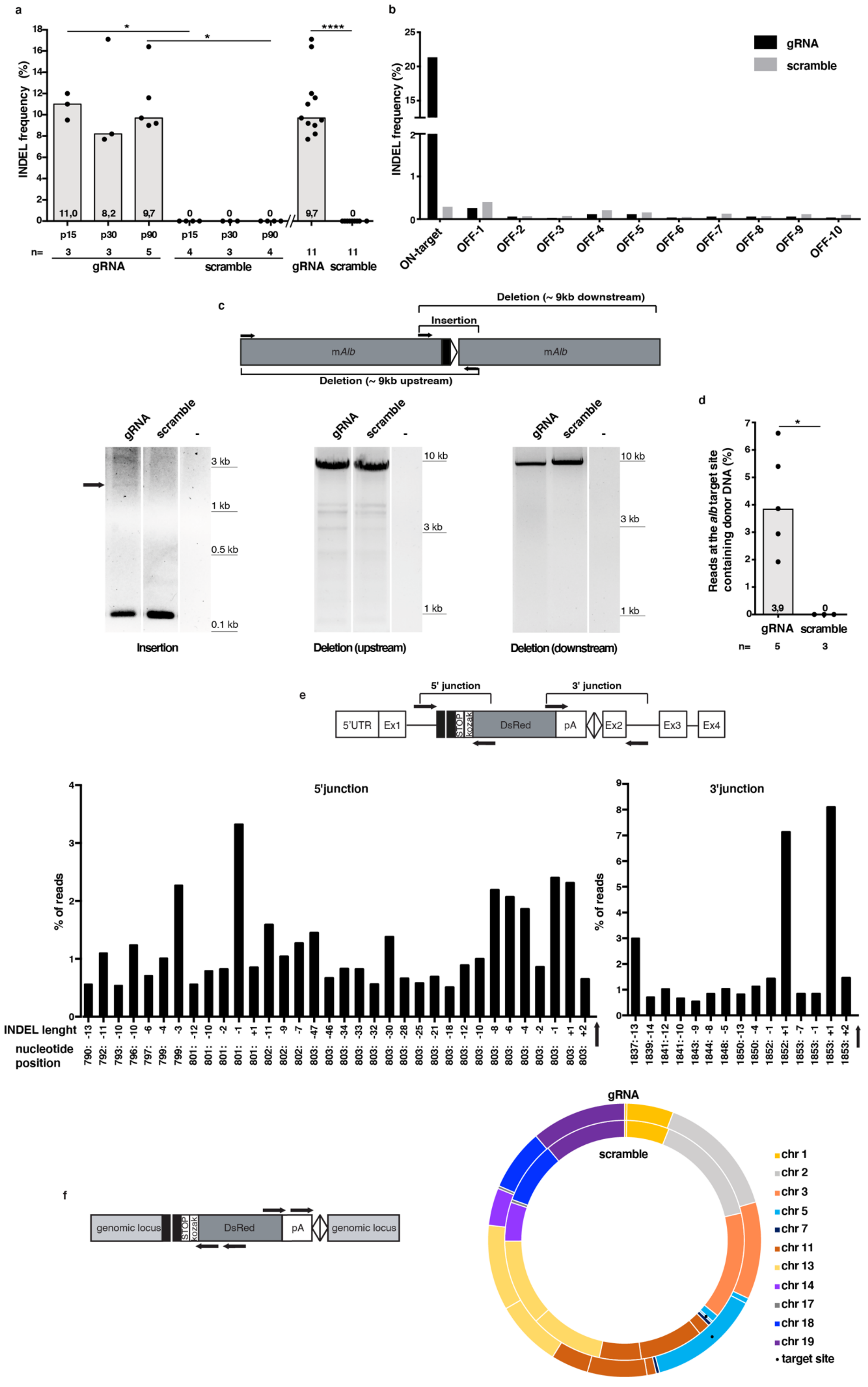
a
INDEL frequency (%)
*
*
****
11,0
8,2
9,7
0
0
0
9,7
0
p15
p30
p90
p15
p30
p90
n=
3
3
5
4
3
4
11
11
gRNA
scramble
gRNA
scramble
b
gRNA
scramble
INDEL frequency (%)
ON-target
OFF-1
OFF-2
OFF-3
OFF-4
OFF-5
OFF-6
OFF-7
OFF-8
OFF-9
OFF-10
c
Deletion (~ 9kb downstream)
Insertion
mAlb
mAlb
Deletion (~ 9kb upstream)
gRNA
scramble
-
3 kb
1 kb
0.5 kb
0.1 kb
Insertion
10 kb
3 kb
1 kb
Deletion (upstream)
10 kb
3 kb
1 kb
Deletion (downstream)
d
Reads at the alb target site containing donor DNA (%)
*
3,9
0
gRNA
scramble
n=
5
3
e
5' junction
3' junction
5'UTR
Ex1
STOP
kozak
DsRed
pA
Ex2
Ex3
Ex4
5'junction
3'junction
% of reads
INDEL lenght
nucleotide position
f
genomic locus
STOP
kozak
DsRed
pA
genomic locus
gRNA
scramble
chr 1
chr 2
chr 3
chr 5
chr 7
chr 11
chr 13
chr 14
chr 17
chr 18
chr 19
• target site

Fig. Supplementary 8. Safety and efficiency of HITI in mouse hepatocytes.

(**a**) TIDE quantification of INDELs on DNAs extracted from treated livers. (**b**) NGS quantification of INDEL frequency at the m*Alb* (ON-target) and at the 10 top off-target (OFF-1-10) sites for the m*Alb*-gRNA, in gRNA- and scramble-treated livers. (**c**) Characterization of large rearrangements by PCR. Schematic depiction of the location of the primers used for PCR amplification of potential large insertions and deletions after SpCas9 cleavage at the m*Alb* locus and relative representative PCR products obtained in 3 gRNA- and 2 scramble-treated samples. The arrow points out to the expected size of the donor DNA integration. (**d**) NGS quantification of HITI DNA in gRNA- and scramble-treated livers. Results are represented as a percentage of total reads. (**e**) Characterization of HITI precision by NGS analysis of HITI junctions. Schematic depiction of the location of the primers used for PCR amplification of HITI junctions and relative INDEL frequency at each position surrounding the cleavage site of either the HITI 5' or 3' junction. Negative and positive numbers represent deletions and insertions, respectively. The black arrow points at the SpCas9-mediated double-strand break. (**f**) NGS characterization of off-target HITI and AAV integration. Schematic depiction of probes designed for NGS analysis of HITI off-targets. The circle plot shows the genomic locations where integration of donor DNA was observed. The bars size represents the relative frequency of integration at each locus. The m*Alb* target site for SpCas9 is highlighted by black asterisks. Integrations with relative frequency higher than 0,2% are represented in the figure.
Results are represented as a single measurement for each mouse (dot, **a**, **d**) and as either median (column, **a**, **b**, **d**) for each group of treatment. *= p<0,05; ****= p<0,0001 (**a**, Wilcoxon rank sum test; **d**, Kruskal-Wallis test followed by the Dunn's test and Wilcoxon test). Further details on statistical analysis, including exact p value, can be found in Methods section. Abbreviations: 5'UTR: 5' untranslated region; m*Alb*: mouse *albumin*; Ex: exon; black rectangles and white triangles represent the two parts of the SpCas9 target sites flanking the SpCas9-induced double strand break; pA: bovine growth hormone polyadenylation sequence; n: number of biologically independent samples.

Supplementary Tables

Table Supplementary 1: gRNAs and scramble sequences used to target SpCas9 to the rhodopsin and albumin loci in different species

gRNA target	gRNA sequence	PAM	Target Strand
mouse *rhodopsin* (m*Rho*)	GCAGCCGCAGTACTACCTGG	CGG	+
pig *Rhodopsin* (p*RHO*)	AGTACTGCGGATACTCAAAG	GGG	-
mouse *albumin* (m*Alb*)	ACAAGAGTGAGATCGCCCAT	CGG	+
scramble	GACTCGCGCGAGTCGAGGAG	NGG	/

Table Supplementary 2. Top 10 ranked OFF-targets of m*Rho* gRNA

Target site	Chromosome	Position	Locus	Sequence	PAM
ON-target	chr6	115908965-115909326	E:Rho	GCAGCCGCAGTACTACCTGG	CGG
OFF-target 1	chr15	59279413-59279435	I:Nsmce2	GCAGCTCCAGTACTACCTGA	TGG
OFF-target 2	chr14	76686275-76686297	I:Tsc22d1	GCAGCAAGAATACTACCTGG	AGG
OFF-target 3	chr11	83927598-83927620	I:Synrg	CCAGCAACAGAACTACCTGG	TGG
OFF-target 4	chr7	135386855-135386877	IG:Mki67-6330420H09Rik	CCAACTGCAGTACTACCTAG	AGG
OFF-target 5	chr16	13222442-13222464	I:Mrtfb	GAAGACACAGTACTACCTGA	GGG
OFF-target 6	chr11	117249472-117249494	I:Septin9	GTGGCCAGAGTACTACCTGG	AGG
OFF-target 7	chr17	47949978-47950000	IG:Usp49-Gm20517/Usp49	GCAAGAGCAGCACTACCTGG	AGG
OFF-target 8	chr3	96931592-96931614	IG:Gja8-Gja5	GCAGCCGCTTCACTAACTGG	AGG
OFF-target 9	chr6	127989925-127989947	I:Tspan9	GCTACCACAGTACTACATGG	TGG
OFF-target 10	chr13	70469132-70469154	IG:4930520P13Rik-1700100L14Rik	GAAGCAGCAGTATAACCTGG	TGG

N.B. Mouse genome reference is GRC mm39. Abbreviations: chr: chromosome; IG: intergenic region; I: gene intron; E: gene exon.

Table Supplementary 3. Predicted genotype frequency following HITI at the m*Rho* locus.

Genotype	Expected outcome	Frequency	Total	
P23H/wild-type	Unmodified		74,0%	
INDEL/wild-type	Therapeutic	7,7%	13,2%	****
HITI/wild-type	Therapeutic	4,3%		
INDEL/HITI	Therapeutic	0,9%		
HITI/HITI	Therapeutic	0,3%		
P23H/INDEL	Presumably no effect		7,7%	
P23H/HITI	No effect		4,3%	
INDEL/INDEL	Deleterious		0,8%	

N.B. ****= p<0,0001 (chi-square test of homogeneity followed by pairwise comparison). Further details on statistical analysis, including exact p value, can be found in Methods section.

Table Supplementary 4. Top 10 ranked OFF-targets of m*Alb* gRNA

Target site	Chromosome	Position	Locus	Sequence	PAM
ON-target	chr5	90609580-90609602	E: *Alb*	ACAAGAGTGAGATCGCCCAT	CGG
OFF-target 1	chr6	52151385-52151407	IG: Gm15050-LOC115490409	ACAAAATTGTAATCGCCCAT	TGG
OFF-target 2	chr9	115681001-115681001	IG: D330037F02Rik-Gadl1	ACAAGAGTGTGAACGCACAT	GGG
OFF-target 3	chr4	45068558-45068580	I: *Fbxo10*	AAAAGACTGAAATCGCCCAA	AGG
OFF-target 4	chr13	86483622-86483644	IG: Cox7c-Gm34585	ACATGTGTGAGACTGCCCAT	GGG
OFF-target 5	chr7	126668845-126668867	IG: Zg16-AI467606	ACAAGTGTGAGAACACCAAT	AGG
OFF-target 6	chr2	143481820-143481842	I: *Pcsk2*	AGAAGAGTCAGGTTGCCCAT	GGG
OFF-target 7	chr1	123282478-123282500	I: *Dpp10*	ACCGGGGTGAGATCACCCAT	GGG
OFF-target 8	chr2	47780320-47780342	IG: Gm13470-Gm13481	ACAAGAGTGAAACTGTCCAT	GGG
OFF-target 9	chr17	3573552-3573574	IG: Tfb1m-Cldn20	AAAAGAATGAGAAAGCCCAT	TGG
OFF-target 10	chr2	96736304-96736326	I: *Lrrc4c*	ACAAGAGTGTGAACGCACAT	GGG

N.B. Mouse genome reference is GRC mm39. Abbreviations: chr: chromosome; IG: intergenic region; I: gene intron; E: gene exon.

Table Supplementary 5: Genome-wide distribution of donor DNA integration

Chromosome	Position	gRNA		scramble		Location
		**Reads (n)**	**Frequency (%)**	**Reads (n)**	**Frequency (%)**	
chr2	31643991-31644145	8399	14,45	5515	15,15	*Prdm12* (I)
chr5	90461602-90461779	7584	13,04	89	0,24	*Alb* (E2)
chr3	80511979-80512222	6573	11,31	5224	14,35	IG
chr19	53754449-53754604	6419	11,04	3982	10,94	*Rbm20* (I)
chr13	56521002-56521159	5757	9,90	4199	11,53	IG
chr13	9045278-9045447	4514	7,76	3555	9,76	IG
chr18	84846054-84846209	4161	7,16	3035	8,34	IG
chr11	69822691-69822844	4023	6,92	3095	8,50	IG
chr1	100362534-100362681	3162	5,44	2076	5,70	*Cntnap5b* (I)
chr11	81618820-81618970	2532	4,36	2034	5,59	*Asic2* (I)
chr14	28471633-28471785	2532	4,36	1893	5,20	*Erc2* (I)
chr11	29765309-29765465	622	1,07	573	1,57	*Eml6* (I)
chr5	27213343-27213489	366	0,63	358	0,98	*Dpp6* (I)
chr7	126595287-126595437	229	0,39	185	0,51	IG
chr17	48108567-48108719	184	0,32	144	0,40	*9830107B12Rik* (I)
chr1	88149778-88149781	160	0,28	105	0,29	*Ugt1a10* (I)
chr15	98842798-98842921	100	0,17	67	0,18	*Kmtd2* (E43)
chr16	52331591-52331593	77	0,13	0	0,00	*Alcam* (I)
chr5	90464373-90464375	73	0,13	0	0,00	*Alb* (I)
chr5	90461994-90461996	69	0,12	0	0,00	*Alb* (I)
chr14	109541111-109541113	63	0,11	0	0,00	IG
chr12	108837963-108837965	55	0,09	40	0,11	IG
chr10	12254869-12254871	54	0,09	43	0,12	IG
chr1	92992918-92993070	51	0,09	42	0,12	IG
chr9	103208889-103208891	48	0,08	0	0,00	*Trf* (E17)
chr5	90455917-90455919	47	0,08	0	0,00	IG
chr10	58536367-58536369	47	0,08	0	0,00	*Ccdc138* (I)
chr5	90508455-90508485	45	0,08	0	0,00	*Afp* (E14)
chr3	48838625-48838627	44	0,08	0	0,00	IG
chr5	148837250-148837252	42	0,07	0	0,00	IG
chr11	88967932-88967934	38	0,07	0	0,00	IG
chr3	97898875-97898877	36	0,06	40	0,11	*LincRNA Gm15999*
chr19	29675316-29675318	34	0,06	0	0,00	IG
chr7	112365276-112365278	0	0,00	43	0,1	IG
chr17	68388269-68388271	0	0,00	32	0,09	*L3mbtl4* (I)
chrX	50797759-50797853	0	0,00	37	0,10	IG

N.B. Mouse genome reference is GRCm38.p6. Abbreviations: chr: chromosome; n: number of reads; IG: intergenic region; I: gene intron; E: gene exon. The number after "E" indicates the exon in which the integration was found.

Supplementary References

1. Venkatesan, A. & Dasgupta, A. Novel fluorescence-based screen to identify small synthetic internal ribosome entry site elements. *Molecular and cellular biology* **21**, 2826–37 (2001).
2. Venkatesan, A. & Dasgupta, A. Novel fluorescence-based screen to identify small synthetic internal ribosome entry site elements. *Mol Cell Biol* **21**, 2826–2837 (2001).
3. Verver, D. *et al.* Non-SMC Element 2 (NSMCE2) of the SMC5/6 Complex Helps to Resolve Topological Stress. *IJMS* **17**, 1782 (2016).
4. Yu, O. M., Miyamoto, S. & Brown, J. H. Myocardin-Related Transcription Factor A and Yes-Associated Protein Exert Dual Control in G Protein-Coupled Receptor- and RhoA-Mediated Transcriptional Regulation and Cell Proliferation. *Mol Cell Biol* **36**, 39–49 (2016).
5. Ishibashi, Y. *et al.* Expression of SOLOIST/MRTFB i4, a novel neuronal isoform of the mouse serum response factor coactivator myocardin-related transcription factor-B, negatively regulates dendritic complexity in cortical neurons. *Journal of Neurochemistry* (2020) doi:10.1111/jnc.15122.
6. Rajan, R. S. & Kopito, R. R. Suppression of wild-type rhodopsin maturation by mutants linked to autosomal dominant retinitis pigmentosa. *J Biol Chem* **280**, 1284–1291 (2005).
7. Humphries, M. M. *et al.* Retinopathy induced in mice by targeted disruption of the rhodopsin gene. *Nat Genet* **15**, 216–219 (1997).
8. Jaissle, G. B. *et al.* Evaluation of the rhodopsin knockout mouse as a model of pure cone function. *Invest Ophthalmol Vis Sci* **42**, 506–513 (2001).
9. Sakami, S. *et al.* Probing mechanisms of photoreceptor degeneration in a new mouse model of the common form of autosomal dominant retinitis pigmentosa due to P23H opsin mutations. *J Biol Chem* **286**, 10551–10567 (2011).
10. Suzuki, K. *et al.* In vivo genome editing via CRISPR/Cas9 mediated homology-independent targeted integration. *Nature* **540**, 144–149 (2016).
11. Suzuki, K. & Izpisua Belmonte, J. C. In vivo genome editing via the HITI method as a tool for gene therapy. *J Hum Genet* **63**, 157–164 (2018).

12. Nakai, H. *et al.* AAV serotype 2 vectors preferentially integrate into active genes in mice. *Nat Genet* **34**, 297–302 (2003).
13. Ng, S. B. *et al.* Exome sequencing identifies MLL2 mutations as a cause of Kabuki syndrome. *Nat Genet* **42**, 790–793 (2010).
14. Cleary, S. P. *et al.* Identification of driver genes in hepatocellular carcinoma by exome sequencing. *Hepatology* **58**, 1693–1702 (2013).
15. Rao, R. C. & Dou, Y. Hijacked in cancer: the KMT2 (MLL) family of methyltransferases. *Nat Rev Cancer* **15**, 334–346 (2015).
16. Arai, E. *et al.* Multilayer-omics analysis of renal cell carcinoma, including the whole exome, methylome and transcriptome. *Int J Cancer* **135**, 1330–1342 (2014).
17. Vila-Carriles, W. H., Zhou, Z. H., Bubien, J. K., Fuller, C. M. & Benos, D. J. Participation of the chaperone Hsc70 in the trafficking and functional expression of ASIC2 in glioma cells. *J Biol Chem* **282**, 34381–34391 (2007).
18. Zhou, Z. H. *et al.* The acid-sensing ion channel, ASIC2, promotes invasion and metastasis of colorectal cancer under acidosis by activating the calcineurin/NFAT1 axis. *J Exp Clin Cancer Res* **36**, 130 (2017).
19. Moss, T. J. *et al.* Comprehensive Genomic Characterization of Upper Tract Urothelial Carcinoma. *Eur Urol* **72**, 641–649 (2017).
20. Zhao, H. *et al.* Cell fate regulation by reticulon-4 in human prostate cancers. *J Cell Physiol* **234**, 10372–10385 (2019).
21. Strassburg, C. P., Manns, M. P. & Tukey, R. H. Differential down-regulation of the UDP-glucuronosyltransferase 1A locus is an early event in human liver and biliary cancer. *Cancer Res* **57**, 2979–2985 (1997).
22. Morimoto, A. *et al.* An HNF4alpha-microRNA-194/192 signaling axis maintains hepatic cell function. *J Biol Chem* **292**, 10574–10585 (2017).
23. Sanders, A. J. *et al.* Importance of activated leukocyte cell adhesion molecule (ALCAM) in prostate cancer progression and metastatic dissemination. *Oncotarget* **10**, 6362–6377 (2019).

24. Lu, X. Y. *et al.* Predicting Value of ALCAM as a Target Gene of microRNA-483-5p in Patients with Early Recurrence in Hepatocellular Carcinoma. *Front Pharmacol* **8**, 973 (2017).

25. Qu, L. S. *et al.* Nine susceptibility loci for hepatitis B virus-related hepatocellular carcinoma identified by a pilot two-stage genome-wide association study. *Oncol Lett* **11**, 624–632 (2016).

26. Sorrentino, A. *et al.* PR/SET Domain Family and Cancer: Novel Insights from the Cancer Genome Atlas. *Int J Mol Sci* **19**, (2018).

27. Zheng, Y. F., Lu, X., Zhang, X. Y. & Guan, B. G. The landscape of DNA methylation in hepatocellular carcinoma. *J Cell Physiol* **234**, 2631–2638 (2019).

28. Reid, A. G. & Nacheva, E. P. A potential role for PRDM12 in the pathogenesis of chronic myeloid leukaemia with derivative chromosome 9 deletion. *Leukemia* **18**, 178–180 (2004).

29. Zhao, X. *et al.* Dipeptidyl peptidase like 6 promoter methylation is a potential prognostic biomarker for pancreatic ductal adenocarcinoma. *Biosci Rep* **40**, (2020).

30. Chandler, R. J. *et al.* Vector design influences hepatic genotoxicity after adeno-associated virus gene therapy. *J Clin Invest* **125**, 870–880 (2015).

31. Donsante, A. *et al.* AAV vector integration sites in mouse hepatocellular carcinoma. *Science* **317**, 477 (2007).

32. Walia, J. S. *et al.* Long-term correction of Sandhoff disease following intravenous delivery of rAAV9 to mouse neonates. *Mol Ther* **23**, 414–422 (2015).

Uncropped images

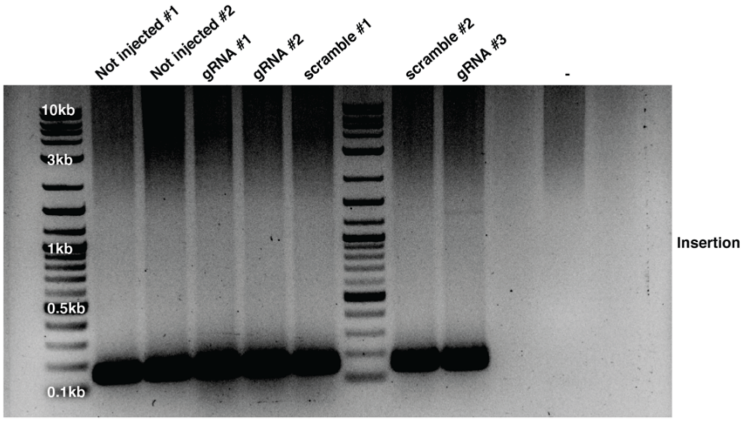

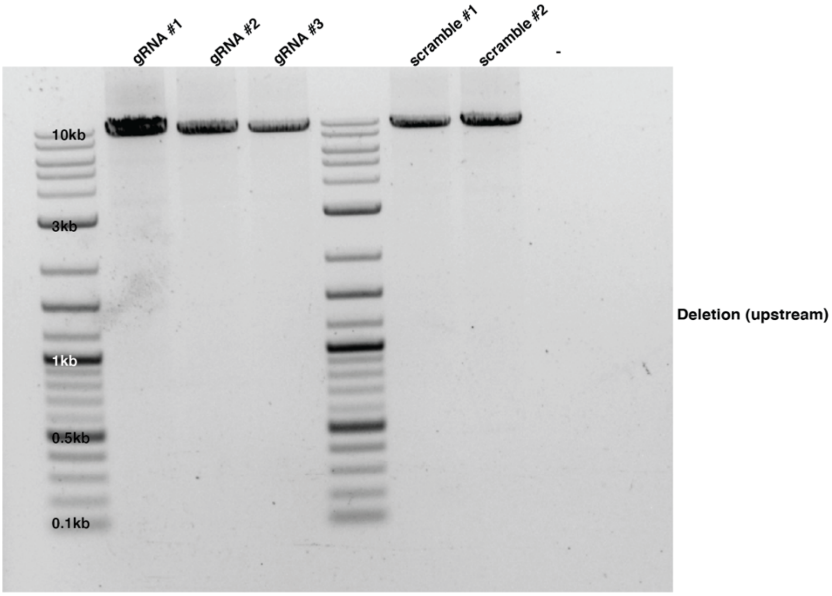

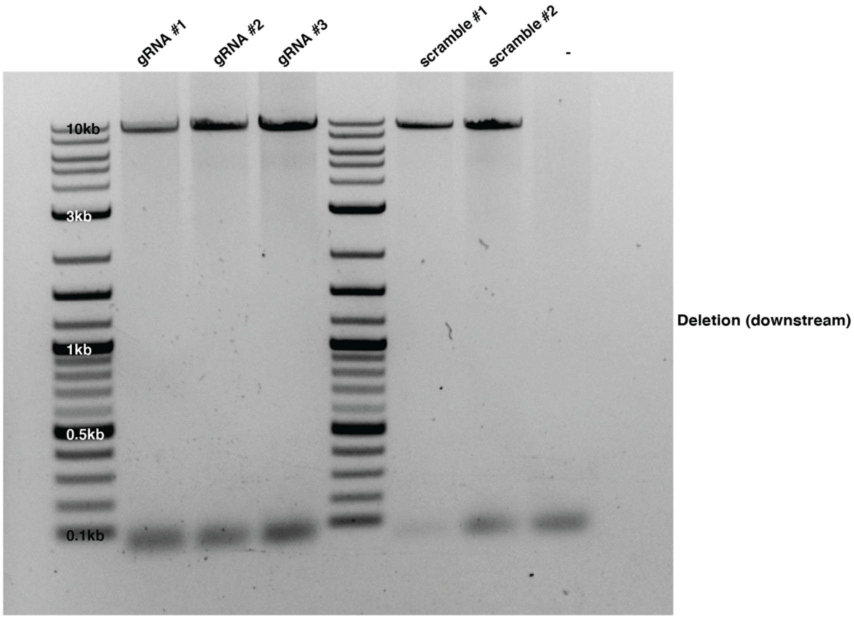

Uncropped images of Supplementary Figure 3c

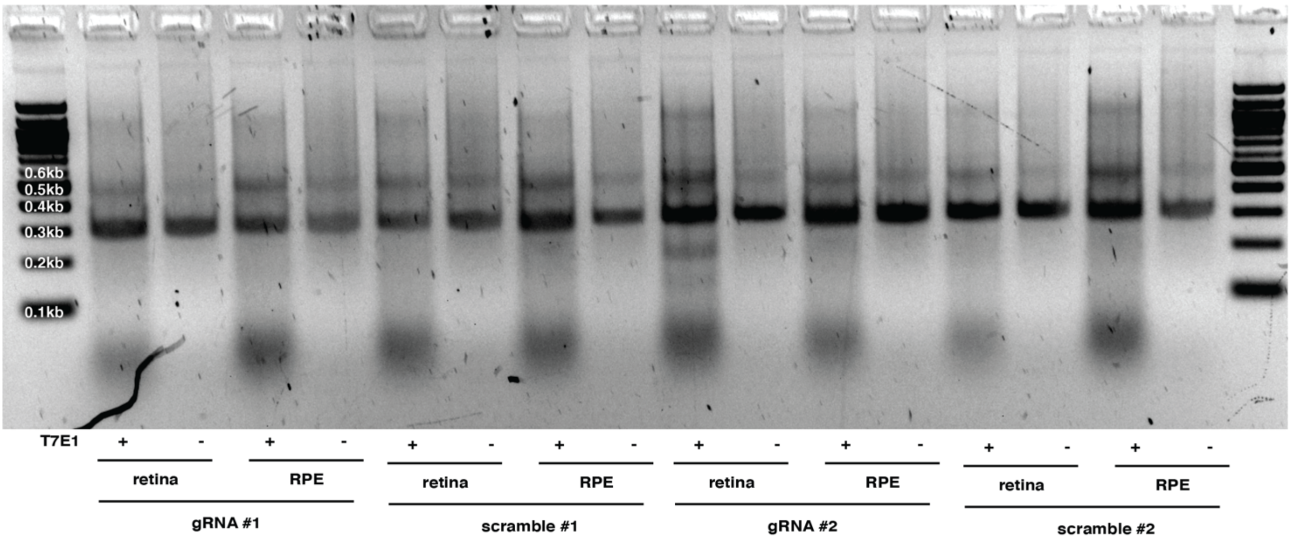

Uncropped image of Supplementary Figure 4a

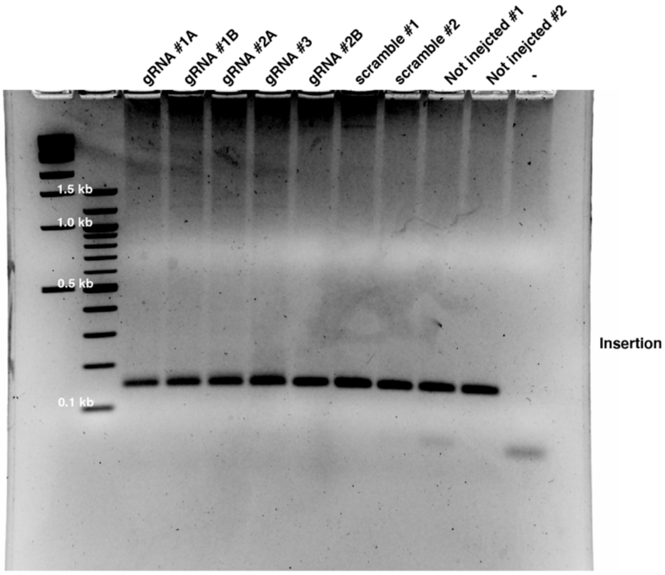

Samples reported as A and B correspond to different rounds of DNA extraction from the same liver.

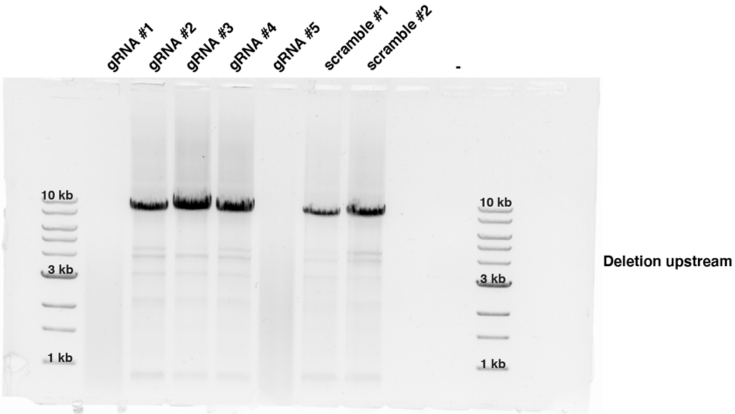

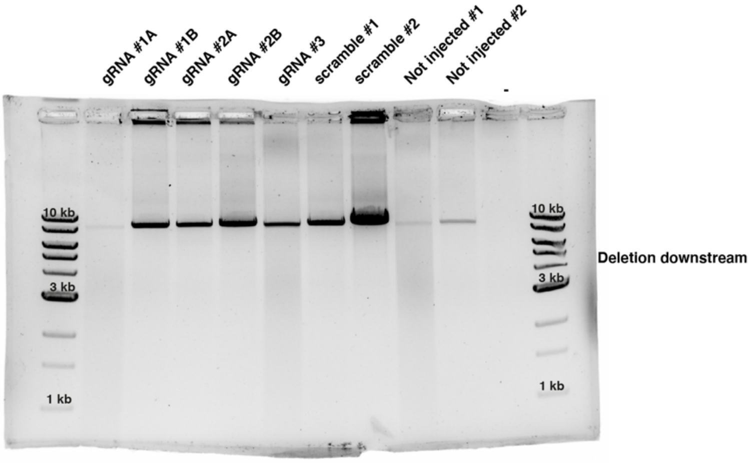

Samples reported as A and B correspond to different rounds of DNA extraction from the same liver.

Uncropped images of Supplementary Figure 8c
