# Supplementary material for: Therapeutic homology-independent targeted integration in retina and liver
Source: Nat Commun. 2022 Apr 12;13:1963. doi: 10.1038/s41467-022-29550-8 (PMC9005519; doi:10.1038/s41467-022-29550-8)
Supplement: Supplementary file 2 — Reporting Summary [file 41467_2022_29550_MOESM2_ESM.pdf]

nature portfolio

Corresponding author(s): Alberto Auricchio

Last updated by author(s): Jan 19, 2022

Reporting Summary

Nature Portfolio wishes to improve the reproducibility of the work that we publish. This form provides structure for consistency and transparency in reporting. For further information on Nature Portfolio policies, see our Editorial Policies and the Editorial Policy Checklist.

Statistics

For all statistical analyses, confirm that the following items are present in the figure legend, table legend, main text, or Methods section.

n/a	Confirmed	
☐	☒	The exact sample size (*n*) for each experimental group/condition, given as a discrete number and unit of measurement
☐	☒	A statement on whether measurements were taken from distinct samples or whether the same sample was measured repeatedly
☐	☒	The statistical test(s) used AND whether they are one- or two-sided<br>*Only common tests should be described solely by name; describe more complex techniques in the Methods section.*
☒	☐	A description of all covariates tested
☐	☒	A description of any assumptions or corrections, such as tests of normality and adjustment for multiple comparisons
☐	☒	A full description of the statistical parameters including central tendency (e.g. means) or other basic estimates (e.g. regression coefficient) AND variation (e.g. standard deviation) or associated estimates of uncertainty (e.g. confidence intervals)
☐	☒	For null hypothesis testing, the test statistic (e.g. *F*, *t*, *r*) with confidence intervals, effect sizes, degrees of freedom and *P* value noted<br>*Give P values as exact values whenever suitable.*
☒	☐	For Bayesian analysis, information on the choice of priors and Markov chain Monte Carlo settings
☒	☐	For hierarchical and complex designs, identification of the appropriate level for tests and full reporting of outcomes
☒	☐	Estimates of effect sizes (e.g. Cohen's *d*, Pearson's *r*), indicating how they were calculated

Our web collection on statistics for biologists contains articles on many of the points above.

Software and code

Policy information about availability of computer code

Data collection	Microscopy images were acquired using LSM-700 (Carl Zeiss), LEICA DM-5000 and Axio Scan Z1 (Carl Zeiss). Facs data were collected using BD-Facs Area 3. Quantitative PCR data were collected by Lightcycler 96 (Roche); PCR data were collected by T100 Thermal Cycler (BioRad); Gel images were acquired using GelDoc XR+ (BioRad); Serum ARSB and GAG data were collected using Infinite F200 (TECAN); ERG data were collected by Ganzfeld stimulator; Visual acuity data were collected by OptoMotry.
Data analysis	The following softwares were used for data analysis: CRISPRessoV2; BWA-SW software (version 0.0.10); deepSNV software package (version 1.30.0, Bioconductor); OptomotryTM software (version VR 1.4.0); Samtools (version 1.3); IGV (version 2.4.17); BWA-MEM software (Illumina); ad-hoc R algorithm; BWA-MEM software (Illumina); bcl2fastq software (version v2.20.0.422, Illumina); Cutadapt software (version 1.9); Image J (Fiji); BD Facs DIVA; Tracking of INDELs by Decomposition softaware; The software codes used for HITI on- and off-target in liver are publicly available at the following links: https://github.com/frankMusacchia/HITI_OffTargetDetection. Custom scripts used for other analysis can be provided upon request according to Next Generation Diagnostic srl policy.

For manuscripts utilizing custom algorithms or software that are central to the research but not yet described in published literature, software must be made available to editors and reviewers. We strongly encourage code deposition in a community repository (e.g. GitHub). See the Nature Portfolio guidelines for submitting code & software for further information.

Data

Policy information about availability of data

All manuscripts must include a data availability statement. This statement should provide the following information, where applicable:

- Accession codes, unique identifiers, or web links for publicly available datasets
- A description of any restrictions on data availability
- For clinical datasets or third party data, please ensure that the statement adheres to our policy

Raw Illumina sequencing data relative to HITI junction characterization and HITI on- and off-target in liver, are deposited on GEO database with the dataset identifier GSE158771 and GSE158759, respectively. Sequencing data relative to SpCas9 gRNA off-targets and HITI efficiency in the retina are deposited on GEO database with the dataset identifier GSE180117 and GSE180875, respectively.

Field-specific reporting

Please select the one below that is the best fit for your research. If you are not sure, read the appropriate sections before making your selection.

☒ Life sciences ☐ Behavioural & social sciences ☐ Ecological, evolutionary & environmental sciences

For a reference copy of the document with all sections, see nature.com/documents/nr-reporting-summary-flat.pdf

Life sciences study design

All studies must disclose on these points even when the disclosure is negative.

Sample size	All sample sizes are described in the legend to each figure. Sample sizes were determined based on previous experience and technical feasibility. No statistical method was used to determine sample size.
Data exclusions	No data were excluded from the analyses
Replication	A minimum of n=3 Independent biological replicates were considered for each experiment, unless otherwise noted in the methods or figure legends. The number and successful attempts are clearly stated through the manuscript for each experiment.
Randomization	In all in vivo studies, right and left eyes were randomly assigned to each treatment group. In addition, in the studies on the disease models, female and male mice were considered equivalent and randomly assigned to treatment groups. All samples were prepared, treated, processed and analyzed in random order.
Blinding	Observers were blind to both genotype and treatment of the animals for in vivo experiments during both collection and data analysis

Reporting for specific materials, systems and methods

We require information from authors about some types of materials, experimental systems and methods used in many studies. Here, indicate whether each material, system or method listed is relevant to your study. If you are not sure if a list item applies to your research, read the appropriate section before selecting a response.

Materials & experimental systems

n/a	Involved in the study	
☐	☒	Antibodies
☐	☒	Eukaryotic cell lines
☒	☐	Palaeontology and archaeology
☐	☒	Animals and other organisms
☒	☐	Human research participants
☒	☐	Clinical data
☒	☐	Dual use research of concern

Methods

n/a	Involved in the study	
☒	☐	ChIP-seq
☐	☒	Flow cytometry
☒	☐	MRI-based neuroimaging

Antibodies

Antibodies used	Custom-made anti-human arylsulfatase B (hARSB) polyclonal antibody (Covalab), Villeurbanne, France). No catalog number is available.
Validation	The antibody was used to detected hARSB by an immune-capture assay. We performed in-house validation of the antibody using appropriate positive and negative controls.

Eukaryotic cell lines

Policy information about cell lines

Cell line source(s)	HEK293 (ATCC, CRL-1573; Hepa 1-6 (ATCC, CRL-1830); Pk15 (ATCC, CCL33)
Authentication	All cell lines were authenticated by microscopic observation
Mycoplasma contamination	All cell lines were tested negative for mycoplasma contamination using EZ-PCR Mycoplasma test kit
Commonly misidentified lines (See ICLAC register)	No commonly misidentified lines were used in this study.

Animals and other organisms

Policy information about studies involving animals; ARRIVE guidelines recommended for reporting animal research

Laboratory animals	Mus musculus [C57BL6J at 4 week-old age or post natal day (p) 2; RHO P23H -/+ at p7; MPS VI at p1-2], both males and females. All mice housed at 23± 1C with light/dark cycles and humidity of 50 ± 5% with food and water available ad libitum.<br>Sus scrofa (Large White female at 3 month-old age).
Wild animals	No wild animals were used in this study
Field-collected samples	No field-collected samples were used in this study.
Ethics oversight	Studies in animals were carried out in accordance with both the Association for Research in Vision and Ophthalmology Statement for the Use of Animals in Ophthalmic and Vision Research and with the Italian Ministry of Health regulation for animal procedures (Ministry of Health authorization number: 588/2019-PR and 147/2015-PR).

Note that full information on the approval of the study protocol must also be provided in the manuscript.

Flow Cytometry

Plots

Confirm that:

- [x] The axis labels state the marker and fluorochrome used (e.g. CD4-FITC).
- [x] The axis scales are clearly visible. Include numbers along axes only for bottom left plot of group (a 'group' is an analysis of identical markers).
- [x] All plots are contour plots with outliers or pseudocolor plots.
- [x] A numerical value for number of cells or percentage (with statistics) is provided.

Methodology

Sample preparation	Hepa1-6, Pk15 and HEK 293 cells were washed once with PBS, detached with trypsin 0.05% EDTA, washed twice with PBS, and resuspended in sorting solution containing: PBS, 5% FBS and 2.5 mM EDTA.
Instrument	BD FACS Aria III
Software	BD FACS Diva software
Cell population abundance	A minimum of either 5000 or 10000 cell/sample 10,000 cells/sample were sorted and analyzed, respectively.
Gating strategy	P1 population was defined based on FSC/SSC parameters. This was further analyzed using appropriate fluorescence filters based on the background fluorescence of negative control cells.

- [x] Tick this box to confirm that a figure exemplifying the gating strategy is provided in the Supplementary Information.
